# Modeling Diffusion of Elongated Particles Through a Narrowing Channel

**DOI:** 10.3390/e27030293

**Published:** 2025-03-12

**Authors:** Anna Strzelewicz, Michał Cieśla, Bartłomiej Dybiec, Monika Krasowska

**Affiliations:** 1 Faculty of Chemistry, Silesian University of Technology, 44-100 Gliwice, Poland; anna.strzelewicz@polsl.pl (A.S.); monika.krasowska@polsl.pl (M.K.); 2 Institute of Theoretical Physics, and Mark Kac Center for Complex Systems Research, Jagiellonian University, 30-348 Kraków, Poland; bartlomiej.dybiec@uj.edu.pl

**Keywords:** tapered channel, narrowing pore, elongated particle, numerical modeling, mean squared displacement, effective subdiffusion, entropic forces

## Abstract

Simulations of the Brownian dynamics of diffusing particles in complex environments provide important information about the characteristics of the medium and the properties of biological processes. Notable examples include the diffusion of ions and macromolecular solutes through channels of varying cross-section, such as pores in biological membranes, living tissues, zeolites, carbon nanotubes, and synthetic porous materials. In these systems, the observed diffusion can exhibit anomalous behavior characterized by a nonlinear increase in the mean squared displacement. In this article, we present a toy model of the diffusion of rod-shaped particles through a narrowing, conical pore with a trapezoidal longitudinal cross-section. Particles of different sizes undergo a random walk due to interactions with the environment (modeled as noise). We study how the diffusion properties change with particle size as a function of pore width. The numerical analysis of diffusion-driven transport through narrowing conical channels reveals its effective subdiffusive, i.e., anomalous, character.

## 1. Introduction

The problem of diffusion in a pore with a varying cross-section arises in various practical and theoretical contexts [1,2,3,4,5,6,7,8]. It plays an important role in technological applications and, in recent years, has attracted the interest of physicists, mathematicians, engineers, and biologists. Notable examples and direct applications include channels in biological membranes, living tissues, zeolites, carbon nanotubes, and artificial pores such as single-nanopore sensors [9,10,11,12,13,14,15,16,17,18]. Spatial confinement, due to the boundary of the channel, can significantly alter the dynamical properties of a system by both limiting the configuration space accessible to diffusing components and increasing the hydrodynamic drag on such components [19,20,21,22,23,24,25].

In a recent study [26], we introduced a simplified model for the diffusion of rigid rod-shaped particles (spherocylinders) through a cylindrical pore and used it to study their translations and rotations as they move through the pore. In particular, we investigated how the geometric properties of the particles affect the type of effective diffusion, the distribution of first passage times, and their orientation within the pore. Our model showed that thicker particles move through the channel more slowly than thinner ones, while their length does not influence the passage time. We also showed that both spherical and rod-like particles undergo normal diffusion, with the first passage time distribution following an exponential asymptotic behavior. The research was inspired by experimental diffusion studies described in detail in [27], in which polystyrene particles with a diameter of 400 nm passed through a single polymer micropore with a length of 11 μm and a diameter of 0.7 μm.

In a more recent work [28], we focused on the kinetics of spherical particles passing through a conical pore restricted by absorbing and reflecting boundaries. We studied the properties of diffusion as a function of particle size (i.e., particle radius) as a function of pore width. Particles of different radii were subjected to a random force, and diffusion quantifiers were measured. In addition to the mean squared displacement, which indicates the anomalous character of the motion, we examined the mean and median of the first passage times. Some additional in silico experiments let us discuss in detail the interplay of entropic forces [29] and boundary conditions influencing the obtained results.

The present study focuses on numerical simulations of the passage of elongated, rigid particles of different sizes through a conical channel. The channel has a narrowing trapezoidal longitudinal cross-section, with an absorbing boundary at the narrow end and a reflecting boundary at the wide end.

To place our results in a broader context, we will briefly mention previous studies of point-like particles performing Brownian motion in channels of varying geometries, such as conical tubes. Berezhkovskii et al. [1] studied diffusion in a long conical tube of varying cross-section, simplifying the three-dimensional problem to a one-dimensional model under local equilibrium assumptions. Their results showed that the mean first passage time (MFPT) differs depending on the direction of diffusion, even for the same channel slope. Subsequently, [8] extended the concept of diffusion in tubes of varying cross-sections by introducing ideas such as “looping” and “direct transitions” to describe particle motion. These notions showed that direct transitions follow free diffusion dynamics while looping is influenced by entropic forces. Other studies have focused on the effective diffusion coefficient $D_{\mathrm{eff}}$ in irregular geometries, such as corrugated or conical channels. For example, Dagdug [30] derived expressions for $D_{\mathrm{eff}}$ in such channels, while Ledesma [22] related spatially dependent diffusion coefficients to macroscopic diffusion in porous media, emphasizing the role of porosity and tortuosity in shaping diffusion behavior. In addition, the concept of entropic particle splitting mechanisms, which separate particles based on size, has received considerable attention. It has been shown that asymmetric channels can rectify entropic fluctuations, reversing particle motion, and allowing control of particle motion through the drift ratchet mechanism. This is particularly useful in microfluidic applications [31]. Finally, Bauer et al. [32] studied the diffusion of finite-size particles in 2D channels with random boundaries. Their simulations revealed anomalous diffusion due to transient particle binding to the channel walls, which further complicates the diffusion process.

In the next section, Model and Methods (Section 2), we describe the model used to simulate the random transition process of individual particles in conical pores, including its underlying assumptions and the simplifications made. Following this, we investigate the diffusion properties in the Results and Discussion section (Section 3). The paper ends with our conclusions (Section 4).

## 2. Model and Methods

**Figure 1:**
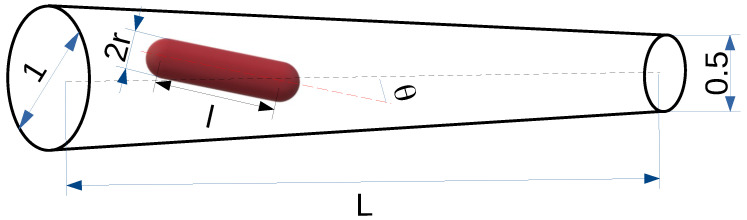
Schematic representation of the experimental setup: a rod-shaped particle diffuses in a conical channel. The length of the channel is *L*. The diameter of the wide end of the channel is equal to the unit length, and the diameter of the narrow end is two times smaller. The particle moving through the channel is a spherocylinder of radius *r* (diameter 2r) and length *l*.

We assume that an elongated particle—see Figure 1—performs a random walk in a radially symmetric conical channel. Initially, a molecule is inserted at the wide end (z=0) of the channel (reflecting boundary), and it continues its motion until absorption at the narrow end (z=L) (absorbing boundary). We assume that only a single molecule is inside the channel at any time.

The motion of the spherocylindrical particle inside the channel has been simulated in the limit of overdamped diffusion. Its position is described by the position of the center of mass and the orientation of the long axis with respect to the channel axis. The single random relocation (translation plus rotation) results from collisions between the spherocylindrical molecule and particles of the environment (thermal bath). Such random collisions produce translations and rotations of the molecule. To simplify the model, we assume that rotational and translational degrees of freedom are decoupled, i.e., the direction of translation and the axis and the direction of rotation do not depend on each other. Within simulations, time is discrete and is measured in the number of displacements. Each displacement consists of a single translation and a single rotation. The displacement of the center of mass of the molecule is calculated as

(1) $$\Delta \vec{x}\left(t\right)=\vec{x}\left(t+1\right)-\vec{x}\left(t\right)=\sigma_{x}{\vec{\xi }}_{t},$$

where ${\vec{\xi }}_{t}=\left[\xi_{x},\xi_{y},\xi_{z}\right]$ is a three-dimensional random vector whose independent components are sampled from the normal N(0,1) distribution. The random vectors ${\vec{\xi }}_{t}$ and ${\vec{\xi }}_{s}$ are independent—i.e., $\langle \xi_{t}^{i}\xi_{s}^{j}\rangle =\delta_{ij}\delta_{ts}$, where $\delta_{ij}$ denotes the Kronecker delta. Equation (1) is consistent with the Euler–Maruyama approximation [33,34] as in the applied units $\sqrt{\Delta t}=1$. To determine the rotation, we first choose a random axis of rotation using a uniform probability distribution over a sphere (centered at the molecule’s center of mass). More precisely, the axis of rotation $\hat{A}$ is determined by the center of the sphere and a random point on the sphere. Afterward, the rotation along such an axis is performed:

(2) $$\Delta \varphi_{t}=\sigma_{\varphi }\eta_{t}.$$

Here, $\Delta \varphi_{t}$ is the rotation angle, and $\eta_{t}$ is again the random number taken from the normal N(0,1) distribution. Analogously to before, $\eta_{t}$ are independent at different times—i.e., $\langle \eta_{t}\eta_{s}\rangle =\delta_{ts}$. Moreover, $\eta_{t}$ and $\xi_{t}^{i}$ are also independent. Finally, the orientation of the molecule is updated using the selected rotation axis and the drawn rotation angle.

The variances $\sigma_{x}$ and $\sigma_{\varphi }$ are proportional to the temperature of the environment (thermal bath). To ensure that the temperature determining translations and rotations is the same, we assume the equipartition of kinetic energy between translational and rotational degrees of freedom of the spherocylinder that relates $\sigma_{x}$ and $\sigma_{\varphi }$ (Appendix C, [26]). Consequently, noise intensities are connected by the following relation:

(3) $$\sigma_{\varphi }\left(\hat{A}\right)=\sigma_{x}{\left({\hat{A}}^{T}M\hat{A}\right)}^{-\frac{1}{2}},$$

where ${\hat{A}}^{T}M\hat{A}$ is the moment of inertia around axis $\hat{A}$; see (Chapter 7.3, [35]). M denotes the moment of inertia matrix of the spherocylinder:

(4) $$M=\left(\begin{array}{ccc} M_{xx} & 0 & 0\\ 0 & M_{yy} & 0\\ 0 & 0 & M_{zz} \end{array}\right),$$

where

(5) $$\begin{array}{ccc} M_{xx}=M_{yy} & = & m_{1}\left(\frac{1}{12}l^{2}+\frac{1}{4}r^{2}\right)+2m_{2}\left(\frac{2}{5}r^{2}+\frac{1}{4}l^{2}+\frac{3}{8}lr\right), \end{array}$$

and

(6) $$\begin{array}{ccc} M_{zz} & = & \left(\frac{1}{2}m_{1}+\frac{4}{5}m_{2}\right)r^{2}. \end{array}$$

The *z*-axis is parallel to the spherocylinder axis, and the *x* and *y* axes are perpendicular to it. *r* is the radius of the hemispheres at each end of the spherocylinder, and *l* is the length of the cylindrical part of the spherocylinder—see Figure 1. All axes intersect at the geometric center of the spherocylinder. $m_{1}$ and $m_{2}$ are the masses of the cylinder and the single hemisphere, respectively. We assume that the particles have uniform density, so the masses $m_{1}$ and $m_{2}$ are related to the characteristic dimensions of the spherocylinder.

(7) $$\frac{m_{1}}{m_{2}}=\frac{3l}{2r}.$$

Condition (7) and the observation that the mass of a homogeneous spherocylinder is $M=m_{1}+2m_{2}$ allow us to calculate all components of its moment of inertia tensor given by (4). According to the relation (3), the tensor of inertia allows us to link the relative dispersions of the translational and rotational parts of the random motion. In the above reasoning, we assume that the mass of the spherocylinder is unit.

The motion of the elongated particles in the conical channel is studied numerically using the Euler–Maruyama algorithm [33,34]. A molecule performs a random walk within the channel consisting of translations—see Equation (1)—and rotations—see Equation (2). To perform a translation, a random displacement with independent components is generated according to $N(0,\sigma_{x})$. The displacement is accepted if it does not lead to a collision with the channel boundaries or to the ejection of the particle through the pore entrance, due to a reflecting boundary at z=0. In other words, the displacements that result in the particle being ejected from the channel through the end where the motion began were rejected. Similarly, to perform the rotation, it is necessary to select an axis of rotation, calculate the moment of inertia of the molecule along that axis, determine the $\sigma_{\varphi }$ parameter, and draw the random angle of rotation from the $N(0,\sigma_{\varphi })$ distribution. The rotation is accepted if it does not cause a collision with channel boundaries. After the translation and rotation have been performed, the time is increased. Finally, it is checked if the particle (its center of mass) is still inside the channel. The whole procedure is continued as long as the molecule’s center of mass is inside the channel (0<z<L). When a particle reaches the exit end of the channel (z⩾L), the first passage time (stopping time) is recorded. The time unit is defined as the number of random steps, each consisting of one translation and one rotation. Note that the number of steps also includes steps with rejected translations or rotations. Furthermore, the whole procedure is averaged over N=1000 repetitions to obtain statistically significant results. In all the simulations, we have used L=15 and $\sigma_{x}=0.05$. See [26] for a detailed description of the molecules’ dynamics.

The diffusive transport through the narrowing channel was analyzed in terms of the mean square displacement (MSD): $\langle x^{2}\left(t\right)\rangle =\langle {\left(\vec{x}\left(t\right)-\vec{x}\left(0\right)\right)}^{2}\rangle$. The diffusion type is then determined by fitting the MSD to the relation

(8) $$\langle x^{2}\left(t\right)\rangle =Dt^{\alpha },$$

where *D* is some constant equal to the diffusion constant when α=1, and α determines the diffusion type. The α=1 case corresponds to standard (normal) diffusion, typical for a Brownian particle. If α<1, the transport is called subdiffusive, which typically means that the motion is hindered by some traps or obstacles, and α>1 corresponds to superdiffusion, which is caused by some internal process that speeds up the random movement [36,37,38,39].

## 3. Results and Discussion

**Figure 2:**
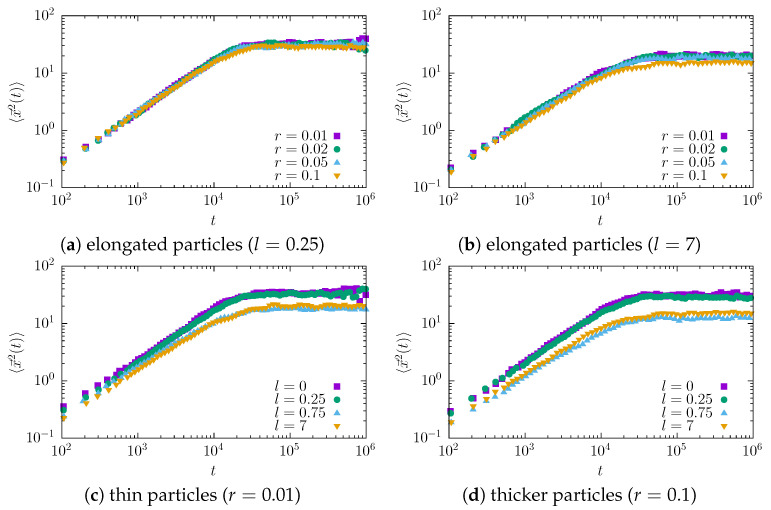
The mean squared displacement (MSD) $\langle {\vec{x}}^{2}\left(t\right)\rangle$ versus time for particles with different lengths and radii: (**a**) length l=0.25 (varying radius); (**b**) length l=7 (varying radius); (**c**) radius r=0.01 (varying length); (**d**) radius r=0.1 (varying length).

The main approach to discriminating the diffusion type is to examine the mean squared displacement. The MSD curves for tracers of different sizes are presented in Figure 2.

**Figure 3:**
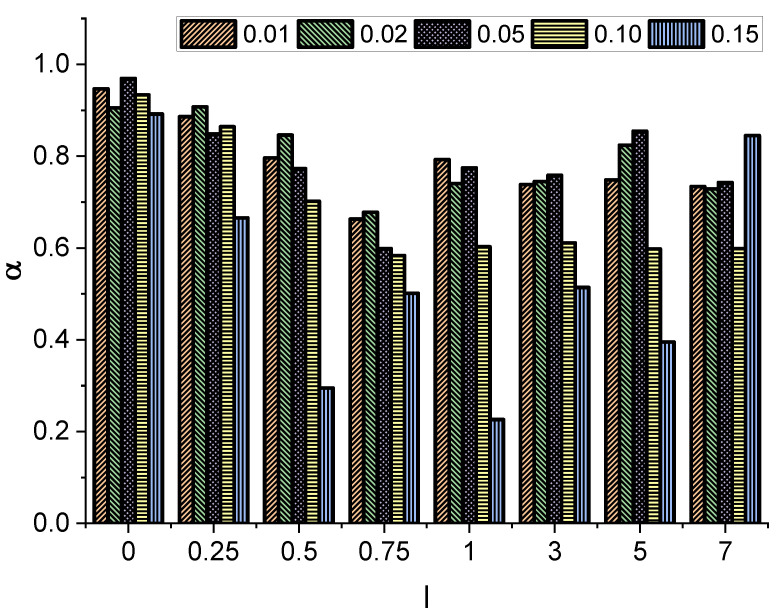
The exponent α, which defines the diffusion type as a function of the particle’s length *l* and radius *r*. The values of α were extracted from fits of Equation (8) to the data obtained from numerical simulations.

In all cases studied, we observed a linear growth (on a log-log scale) of the MSD, which reaches a plateau due to the finite length of the channel and the fact that a tracer that has already reached the narrow end of the channel is no longer included in the average. The observed growth is slower for thicker and especially for longer molecules. To quantify this systematically, we fit the measured data to the relation given by Equation (8) to determine the exponent α. The fitting only considered points obtained before any of the tracers traveled through the entire channel. Specifically, we found the minimum time $t_{min}$ at which one of the tracers (the “fastest” one) reached the narrow end of the channel. Then, for fitting purposes, we used only the data $\langle x^{2}\left(t\right)\rangle$ from the range $t\in [0.01t_{min},t_{min}]$. The fit was based on the least squares method applied to the power function. The results are shown in Figure 3.

Interestingly, in all cases, the obtained value of the exponent α is less than 1, indicating effective subdiffusion. Furthermore, the exponent α decays with increasing molecule length *l*. At l≈0.75, the minimum value of α is recorded. The further increase in the molecule length does not significantly affect the effective diffusion type, as longer particles start to align and move diffusely along the channel, practically without rotations.

The subdiffusive type of observed motion is in contrast to previous observations in a similar system but with the cylindrical channel, where normal diffusion was observed regardless of the tracer size [26]. Such an observation, i.e., normal diffusion, is consistent with earlier theoretical considerations that suggested normal diffusion as long as the slope of the cone is constant [19,20,29]. It has also been shown that for the three-dimensional, rotationally symmetric narrowing channel, the quality of the approximation provided by the Fick–Jacobs equation [40], describing diffusion in a tube of varying cross-section, can be improved by assuming that the effective diffusion coefficient depends on the radius of the channel. For example, in Ref. [29],

(9) $$D_{\mathrm{eff}}≃\frac{D}{1+\frac{1}{2}R^{\prime }\left(z\right)}$$

was suggested, while in Refs. [19,20],

(10) $$D_{\mathrm{eff}}=\frac{D}{\sqrt{1+{\left[R^{\prime }\left(z\right)\right]}^{2}}},$$

where $R^{\prime }\left(z\right)$ is the derivative of the channel radius at position *z*. The general solution of the Fick–Jacobs equation can be found in Ref. [41]. On the one hand, the very similar case of a point-like particle in a conical channel with reflecting and adsorbing boundaries was studied numerically in Ref. [1]. However, in [1], the authors focused on the mean first passage time without studying the effective type of diffusive motion. On the other hand, the recent numerical study [28] on motion of spherical particles in a narrowing channel revealed effective subdiffusion, while for the widening channel effective superdiffusion was observed. In contrast, far from the boundaries, the diffusion appeared to be normal [28]. In conclusion, the observed slowing of the diffusion—see Figure 3–has to be attributed to entropic forces facilitating the motion to the left [19,29,42], since for the cylindrical channel, only standard diffusion was observed [26]. Finally, the specific setup considered here, i.e., implicit boundary conditions and directional (noise induced) motion towards the narrow end, are also important in inducing the effective subdiffusion.

**Figure 4:**
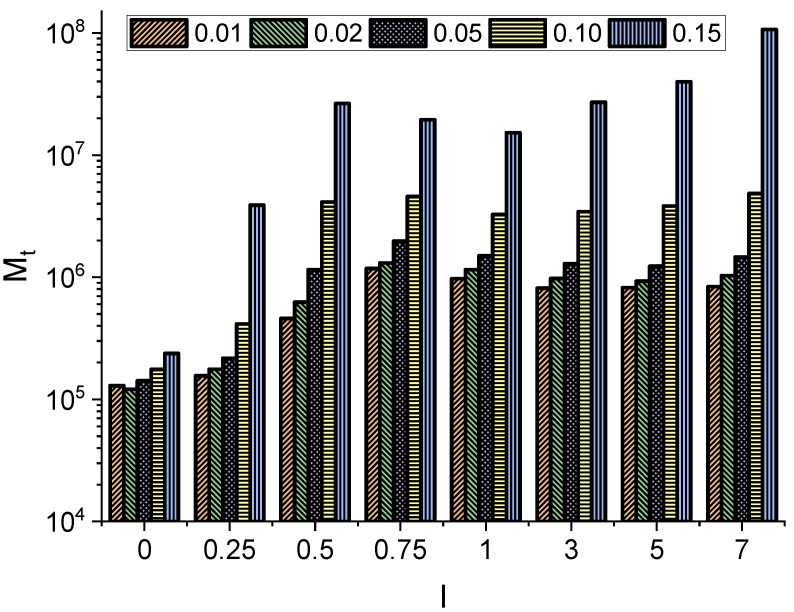
The median ($M_{t}$) of the first passage time as a function of varying particle length and radius, i.e., length l∈{0,0.25,0.5,0.75,1,3,5,7} and radius r∈{0.01,0.02,0.05,0.1,0.15}.

Particle kinetics can be further characterized by the first passage time, which measures the time it takes for the particle to leave the channel for the first time. Figure 4 shows the median ($M_{t}$) of the first passage time, which is the robust characteristic of random variables, i.e., first passage times characterizing the escape process. From Figure 4, it can be seen that longer and thicker molecules need more time to pass through the channel, as the increasing particle size leads to the rejection of many displacements. Here, for molecules longer than 0.75, it can be seen that the median of the first passage time is not very sensitive to the further increase in the molecule length. In contrast, for a fixed *l*, the median $M_{t}$ depends strongly on the radius *r* of the molecule.

**Figure 5:**
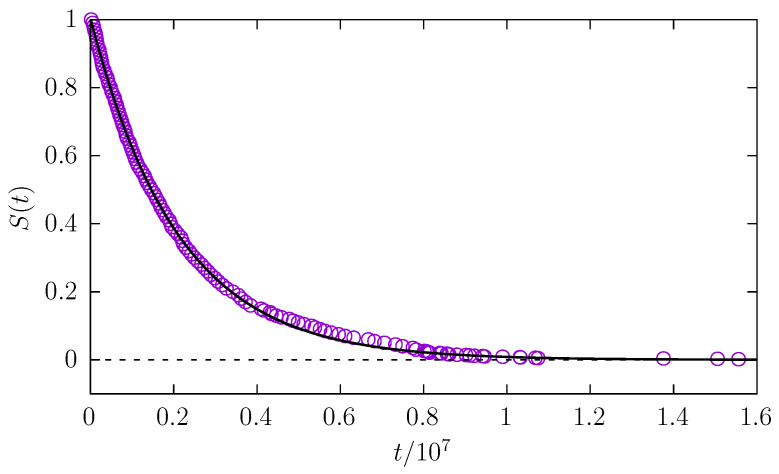
The exemplary survival probability (S(t)) for a particle of length l=7 and radius r=0.05. Points represent the results of simulations, while the line presents the exponential fit $S\left(t\right)\approx 1.005\exp\left[-4.7\cdot 10^{-7}\times t\right]$.

**Figure 6:**
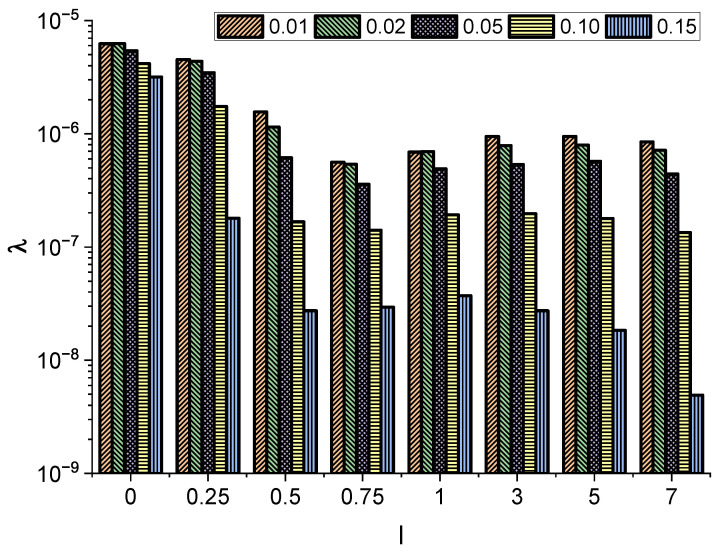
The exponent λ characterizing the decay of the survival probability for a particle of length l∈{0,0.25,0.5,0.75,1,3,5,7} and radius r∈{0.01,0.02,0.05,0.1,0.15}.

In addition to the median $M_{t}$ of the first passage time, we measured the survival probability S(t), i.e., the probability that a molecule is still in the channel at time *t*. Figure 5 shows the example survival probability along with the exponential fit S(t)=Aexp(−λt). The exponent λ measures the effectiveness of the escape kinetics. A faster escape results in larger values of λ, while a slowing of the escape kinetics results in the decay of λ. From Figure 6, it is possible to see how the fitted exponent λ varies with molecule length and radius. Generally, for a fixed molecule length, thicker particles require more time to leave the channel. However, the dependence on molecule length is more intriguing. Analogous to α (the exponent characterizing the diffusion type) and $M_{t}$ (the median of the first passage time), there is a threshold molecule length beyond which the exponent λ does not decrease with a further increase in the particle length. This suggests that after reaching a certain critical length, a particle aligns along a channel and the further motion is not very sensitive to the increase in molecule length. At the same time, the dynamics of a particle are sensitive to its radius, since for fixed *l* the exponent λ decays with increasing radius of the molecule.

**Figure 7:**
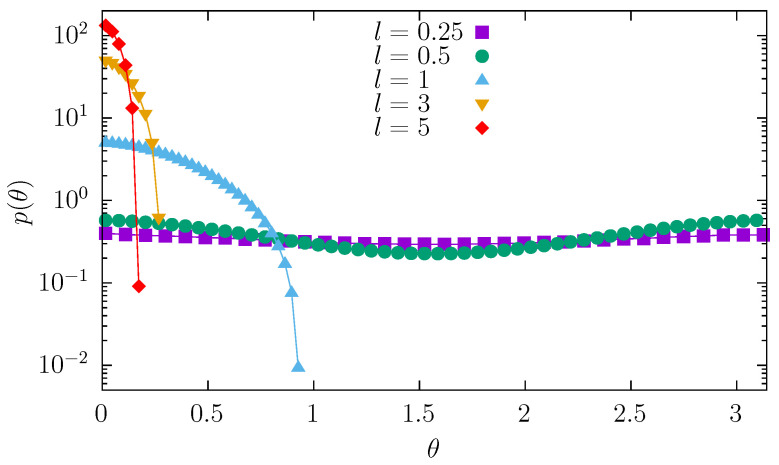
Histogram showing particle orientation distribution, i.e., angle θ between particle axis and channel axis. Various curves correspond to different molecules’ lengths l∈{0.25,0.5,1,3,5} with fixed radius r=0.1.

While moving along the channel, the molecule changes its orientation and position. The orientation of the particle can be characterized by the angle θ measured between the channel axis and the molecule axis. The dependence of the orientation of the molecule is shown in Figure 7. A very short molecule can easily change its orientation. Consequently, the angle distribution is practically uniform. As the length of the molecule increases, it is more difficult to rotate the molecule and more challenging to keep it perpendicular to the channel axis. Consequently, the p(θ) density starts to diminish at θ=π/2. Finally, sufficiently long molecules are practically unable to rotate and remain parallel to the channel axis. In addition, the decreasing width of the channel makes rotation even more difficult. The problem of rotation is loosely related to translations of the molecule.

**Figure 8:**
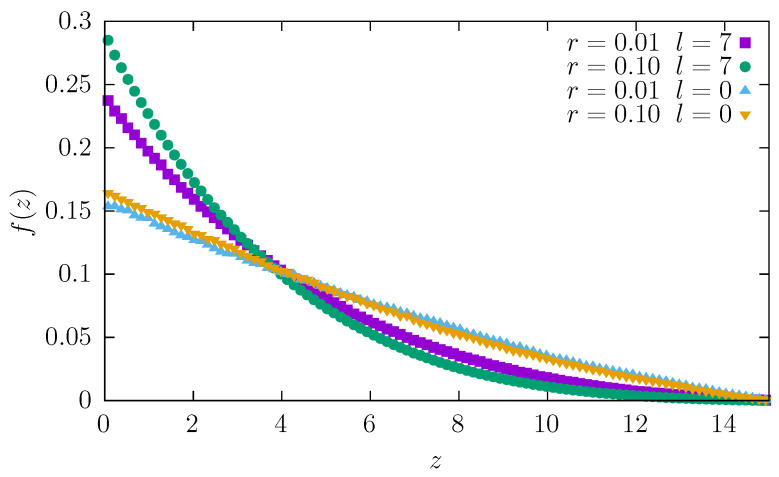
The fraction of time f(z) spent at a given position *z* while passing through the channel. Tracer parameters are r=0.01 or r=0.1, and l=0 or l=7.

To examine in detail how the particle moves through the channel, we recorded a heatmap of the tracer position. In Figure 8, we plot the histogram of the *z* component of the particle position, which changes from 0 at the wider end of the channel to *L* at the narrower end (0<z<L).

The plot shows that a tracer spends most of its time near the wider end because entropic forces push it away from the second (narrow) end. As expected, the effect is more pronounced for longer and thicker particles, for which these forces are stronger. Note that the fraction of time spent at a given position—f(z) shown in Figure 8—is equal to the probability density p(z) of finding a tracer at *z*. Therefore, the entropy, which is proportional to the logarithm of the number of microstates, is proportional to p(z) and as well as to f(z), which also decreases as *z* grows. The entropic force $F_{S}\left(z\right)$ [43,44] is proportional to the gradient of the entropy; hence,

(11) $$F_{S}\left(z\right)∼\frac{d\ln f(z)}{dz}=\frac{f^{\prime }\left(z\right)}{f(z)}.$$

The tracer is thus pushed to the left towards the wider end of the channel, slowing down the diffusion and increasing the fraction of time spent at wider parts of the channel.

## 4. Conclusions

Diffusion is one of the most ubiquitous processes in nature. Effective diffusion approximation can be applied to systems where an exact description is impossible due to the unimaginable number of interactions of the test particle with other molecules. The approximate, effective description of diffusive processes can be provided by the concept of noise, which is used to simplify complicated interactions with the environment. The properties of diffusion are affected by the type of noise, the interactions with obstacles, and the boundaries of the domain of motion.

The present study was designed to determine the influence of particle shape on diffusion processes in narrowing pores. Particle motion was modeled with both translational and rotational degrees of freedom. The results of this investigation show that the confinement of the particles and their interactions with the walls of the narrowing channel are responsible for the emergence of effective subdiffusion. The observed dynamics are sensitive to the size of the molecules; however, for the rigid particles studied here, there is a critical spherocylinder length, resulting in a lack of significant sensitivity to particle length. At the same time, the sensitivity of the results obtained to the radius of the spherocylinder is preserved for all lengths of molecules.

The random motion of the spherocylindrical tracers through the narrowing channel indicates a subdiffusive character. This observation is in contrast to the previous result for a cylindrical channel, where normal diffusion was observed regardless of tracer size [26]. Movement is most hindered for particles of moderate length, which can rotate and align perpendicular to the channel axis. Such orientation causes additional resistance from the channel boundaries. This effect is smaller for shorter particles and does not occur for longer particles because they align along the channel axis. The dependence of the survival probability on time is exponential for all particle sizes, indicating that the rate of tracers leaving the channel in a given time is constant. Further analysis shows that, as expected, this rate is higher for smaller particles.

## Data Availability

The original data presented in the study are openly available in the RODBUK Cracow Open Research Data Repository available at https://doi.org/10.57903/UJ/JMNNFB.

## Abbreviations

The following abbreviations are used in this manuscript:

MFPT	Mean First Passage Time
MSD	Mean Squared Displacement
D	Diffusion Coefficient
